# Sepiapterin reductase: Characteristics and role in diseases

**DOI:** 10.1111/jcmm.15608

**Published:** 2020-07-30

**Authors:** Yao Wu, Peng Chen, Li Sun, Shengtao Yuan, Zujue Cheng, Ligong Lu, Hongzhi Du, Meixiao Zhan

**Affiliations:** ^1^ Jiangsu Key Laboratory of Drug Screening China Pharmaceutical University Nanjing China; ^2^ Department of Neurosurgery The Second Affiliated Hospital of Nanchang University Nanchang China; ^3^ Interventional Radiology Center Zhuhai People's Hospital Zhuhai Hospital Affiliated with Jinan University Zhuhai China; ^4^ School of Pharmacy Hubei University of Chinese Medicine Wuhan China

**Keywords:** cancer, non‐enzymatic activity, sepiapterin reductase, SPR deficiency, tetrahydrobiopterin

## Abstract

Sepiapterin reductase, a homodimer composed of two subunits, plays an important role in the biosynthesis of tetrahydrobiopterin. Furthermore, sepiapterin reductase exhibits a wide distribution in different tissues and is associated with many diseases, including brain dysfunction, chronic pain, cardiovascular disease and cancer. With regard to drugs targeting sepiapterin reductase, many compounds have been identified and provide potential methods to treat various diseases. However, the underlying mechanism of sepiapterin reductase in many biological processes is unclear. Therefore, this article summarized the structure, distribution and function of sepiapterin reductase, as well as the relationship between sepiapterin reductase and different diseases, with the aim of finding evidence to guide further studies on the molecular mechanisms and the potential clinical value of sepiapterin reductase. In particular, the different effects induced by the depletion of sepiapterin reductase or the inhibition of the enzyme suggest that the non‐enzymatic activity of sepiapterin reductase could function in certain biological processes, which also provides a possible direction for sepiapterin reductase research.

## 1 INTRODUCTION

**Figure 1:**
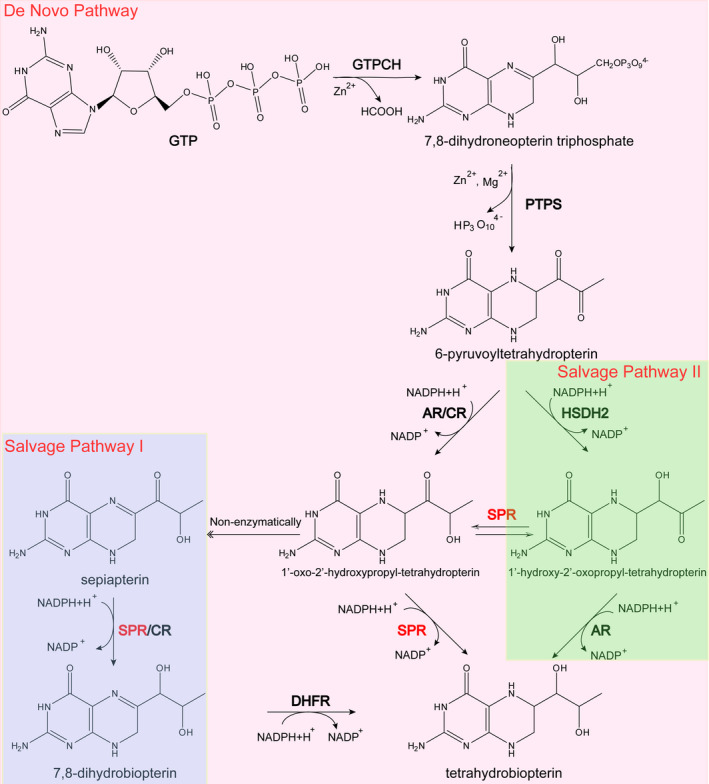
The biosynthetic pathway of tetrahydrobiopterin: de novo pathway from GTP and two other salvage pathways from sepiapterin and 1′‐hydroxy‐2′‐oxopropyl‐tetrahydropterin, respectively. DHFR: dihydrofolate reductase

Tetrahydrobiopterin (BH_4_, 6R‐L‐erythro‐5,6,7,8‐tetrahydrobiopterin) is a key cofactor for a set of enzymes, including nitric oxide synthases (NOSs), aromatic amino acid hydroxylases and alkylglycerol monooxygenase. Consequently, BH_4_ is associated with various biological processes and pathological states, including monoamine neurotransmitter formation, immune response, cardiovascular function, endothelial dysfunction and cancer.^1^, ^2^, ^3^, ^4^, ^5^, ^6^, ^7^ As shown in Figure 1, the primary enzymes involved in the pathway for the biosynthesis of BH_4_ include GTP cyclohydrolase I (GTPCH), 6‐pyruvoyltetrahydropterin synthase (PTPS) and sepiapterin reductase (SPR).^8^, ^9^ With regard to the regulation of BH_4_ biosynthesis, the major step controlling the pathway is GTP cyclohydrolase I. Besides, sepiapterin reductase takes part in the NADPH‐dependent reduction of sepiapterin and 1’‐oxo‐2’‐hydroxypropyl‐tetrahydropterin, which are involved in the salvage and de novo synthetic pathways of tetrahydrobiopterin. Therefore, SPR is also essential for tetrahydrobiopterin biosynthesis.

Due to the important role of BH_4_ in various biological processes, SPR is inferred to be required for many functions at the in vitro and in vivo levels. Therefore, tremendous efforts have been made in attempts to unfold the molecular basis of the function of SPR. Based on the analysis of crystals and nuclear magnetic resonance (NMR) studies, the structure of SPR has been solved for various species. Moreover, gene cloning, recombinant expression and mutagenesis studies have enabled people to understand the biological functions and roles of SPR in different diseases. Meanwhile, many compounds have been identified in relation to SPR, which has provided potential therapeutics for brain dysfunction, cardiovascular disease and cancer. However, there are still many questions unresolved. In particular, there are different effects on biological processes induced by SPR inhibitors and SPR knockdown, for example effects on nitric oxide (NO) generation. Therefore, the main focus in this review will be on the structure, function, distribution and regulation of sepiapterin reductase. In addition, the relationship between SPR and different diseases will also be summarized to determine the possible clinical value of SPR.

## 2 STRUCTURE OF SEPIAPTERIN REDUCTASE

**Figure 2:**
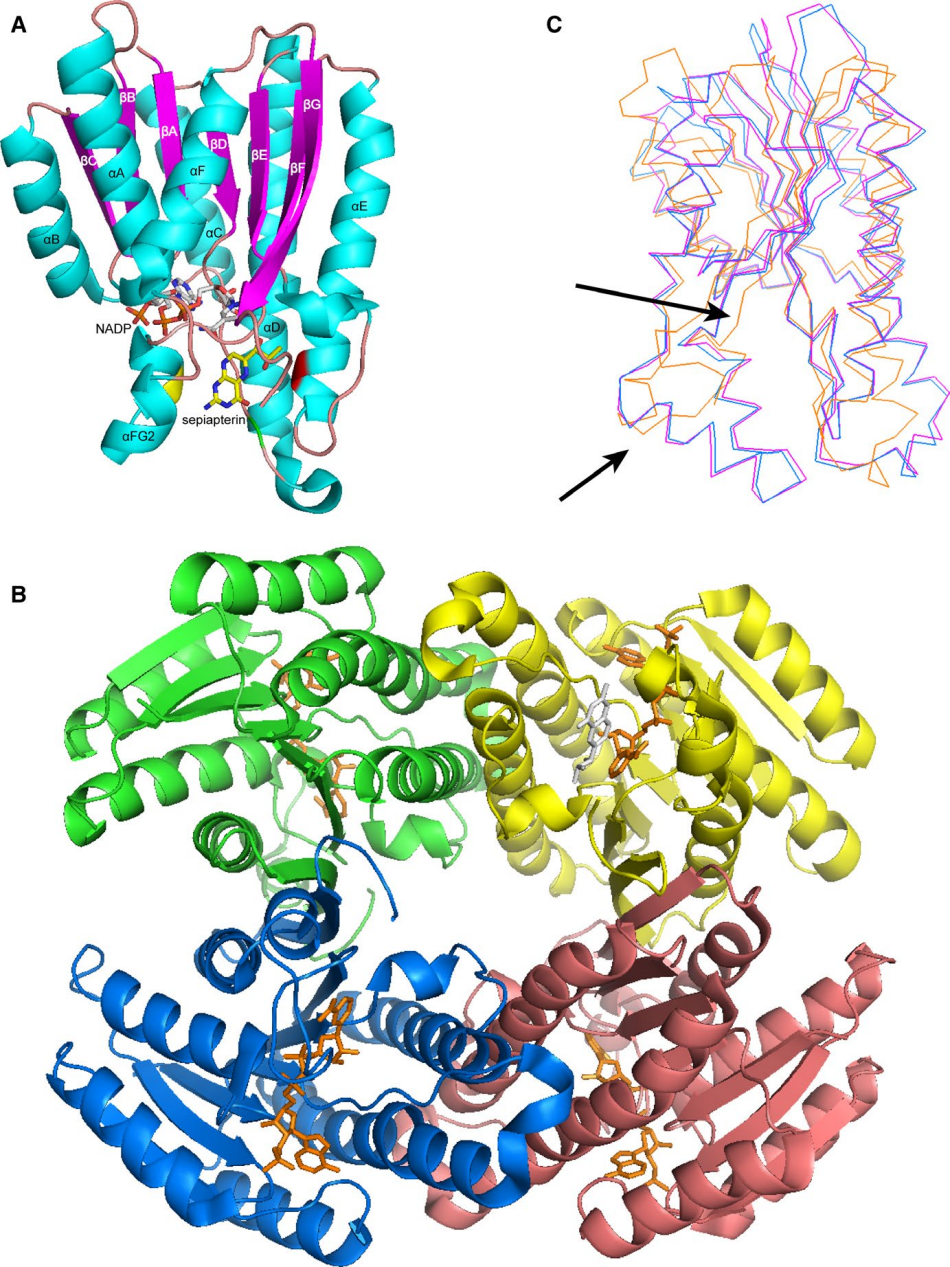
The overall structure of CT‐SPR. A, Stereoview of the ribbon representation of CT‐SPR monomer which binds with NADP and sepiapterin (PDB code 2BD0). β‐Strands and α‐helices are labelled in alphabetical order from the N terminus; three amino acid residues that are essential for catalysis and substrate binding have been labelled by a different colour (F99 is in green; S158 is in red; W196 is in yellow) (B) Ribbon representation of a CT‐SPR tetramer formed by two dimers in the asymmetric unit. NADP is in orange; sepiapterin is in white. C, The comparison of SPR monomer from *C tepidum* (orange), mouse (blue, PDB code 1SEP) and human (magenta, PDB code 4Z3K). Arrows show the largest differences among the three structures

Sepiapterin reductase has structural similarity to that of members of the NADP(H)‐preferring short‐chain reductase family, which contain a strictly conserved Tyr‐Xaa‐Xaa‐Xaa‐Lys sequence motif. Specifically, SPR exists in solution as a homodimer composed of two subunits with a molecular mass calculated to be approximately 28 kDa.^10^, ^11^, ^12^, ^13^ The crystal structures of SPR (Figure 2) indicate that 261 amino acids of each monomer fold into a single domain with an α/β‐structure. A seven‐stranded anti‐parallel oriented β‐sheet in the centre of the molecule is sandwiched by two arrays of three α‐helices. Six of these strands could form a classic nicotinamide‐binding motif composed of βαβ units. The association of two monomers into the active homodimeric SPR leads to the formation of a four‐helix bundle (helices αE and αF of each monomer).^14^, ^15^

In addition to human beings, this enzyme has been identified in rats, mice, monkeys, *Chlorobium tepidum* (*C tepidum*), and *Drosophila*. In addition, the amino acid sequence comparison derived from cDNAs reveals high homology among these different species;^16^, ^17^ for example, the sequence of human SPR (hSPR) shows 74% identity with the rat sequence,^10^ which suggests that the tertiary and quaternary structures of SPR may be conserved. Typically, the NADP(H) binding domain and the positions of active sites show high similarity. Remarkable differences among SPR from various sources are present in the substrate‐binding regions around the active sites. For example, the SPR from *C tepidum* (CT‐SPR) contains a shorter loop and longer C‐terminal extension compared to mouse SPR and hSPR, resulting in diverse stereospecific catalysis reactions.^15^

Furthermore, the active sites have been explored by constructing truncation mutants and through the use of site‐directed mutagenesis. Unlike the N‐terminal A‐X‐L‐L‐S sequence of other BH_4_‐requiring aromatic amino acid hydroxylases, the region of SPR is speculated to preferably act as the coenzyme NADP(H) binding site.^18^ Amino acid residues including Ser‐158, Tyr‐171 and Lys‐175 play an important role in proton transfer and stabilization for the carbonyl group of substrates, according to the SPR crystal structure and kinetic properties of site‐directed mutants. In addition, the catalytic activity could not be detected in the double‐point mutant, SPRY171V + S158D, as opposed to the single‐point mutant, suggesting that the remaining residue might function alone and show low activity if either of the important residues is mutated.^18^, ^19^ However, Trp‐196 and Phe‐99 are indispensable for substrate binding in CT‐SPR because of the swivelled sepiapterin binding mode.^20^ In brief, all of these revelations regarding the structure of SPR make it possible to explore its function and develop therapeutic strategies.

## 3 BIOLOGICAL FUNCTIONS

It is well known that sepiapterin reductase acts as a key enzyme in the biosynthetic pathway of tetrahydrobiopterin cofactor. As shown in Figure 1, sepiapterin reductase takes part not only in the salvage biosynthetic pathway of tetrahydrobiopterin, in which it catalyses the NADPH‐mediated reduction of sepiapterin to dihydrobiopterin,^21^, ^22^ but also in the de novo synthetic pathway, in which it catalyses the conversion of 1′‐oxo‐2′‐ hydroxypropyl‐BH_4_ to BH_4_.^23^, ^24^, ^25^, ^26^ Moreover, another new activity of SPR has been identified, namely ‘lactoyl‐BH_4_ isomerase’ activity, which converts 1′‐hydroxy‐2′‐oxopropyl‐BH_4_ into 1′‐oxo‐2′‐ hydroxypropyl‐BH_4_ independently of NADPH.^27^, ^28^, ^29^ Additionally, many non‐pteridine derivatives, including quinones, for example p‐quinone and menadione; other vicinal dicarbonyls, for example methylglyoxal and phenylglyoxal; monoaldehydes, for example p‐nitrobenzaldehyde; and monoketones, for example acetophenone, acetoin, propiophenone and benzylacetone, are sensitive as substrates of SPR.^30^, ^31^ Furthermore, it has been demonstrated that carbonyl reductases (CR) and aldose reductases (AR), which are primarily active in the liver, could take the place of SPR by an alternative pathway in the biosynthesis of BH_4_. Specifically, CR could also catalyse the conversion of sepiapterin, and AR serves a catalytic function in converting 1′‐hydroxy‐2′‐oxopropyl‐BH_4_ to BH_4_.^32^, ^33^, ^34^ Furthermore, the discovery of patients with sepiapterin reductase deficiency (SPD) who show normal urinary excretion of pterins supports the proposal that BH_4_ biosynthesis from 6‐pyruvoyltetrahydropterin could be compensated by carbonyl and/or aldose reductases in the case of complete hSPR defect and suggests the possible role of the non‐enzymatic activity of SPR in the disease.

The important role of SPR in the biosynthesis of nitric oxide has also been studied based on the conclusion that tetrahydrobiopterin is a limiting factor of nitric oxide generation. According to these results, SPR inhibitors could abolish cytokine‐induced NO production in various cell types,^35^, ^36^, ^37^, ^38^ such as murine macrophages and endothelial cells, but do not affect the constitutive level of NO.^37^, ^38^ Nevertheless, knockdown or overexpression of SPR could significantly affect the constitutive level of NO both in vitro and in vivo.^39^ One hypothetical reason for this controversial conclusion is the function of the non‐enzymatic activity of SPR in the regulation of NO generation. On the other hand, SPR is also involved in oxidative stress. It has been reported that SPR inhibitors could prevent the protective effect of sepiapterin against cell injury induced by H_2_O_2_ in endothelial cells.^40^ The knockout of SPR could impair mitochondrial function and increase the susceptibility of *Dictyostelium discoideum* Ax2 to oxidative stress.^41^ Meanwhile, these results have been proven by the SPR enzymatic inhibitor ‐SPRi3 in CD^4+^ T cells.^42^ However, site‐directed mutagenesis of SPR indicates that mutation of Asp‐257 to histidine abolished sepiapterin reduction activity but had minimal effects on reactive oxygen species production,^43^ suggesting the biological function of the non‐enzymatic activity of SPR (Table 1). Moreover, our published study indicated that SPR could promote hepatocellular carcinoma progression via FoxO3a/Bim signalling in a non‐enzymatic manner, while its enzymatic activity might have no effect on hepatocellular carcinoma development.^44^ Overall, SPR plays a key role in different biological processes, and these effects might be related not only to its enzymatic activity but also to its non‐enzymatic function. Increasing studies prove that many enzymes are involved in tumour progression independent of their catalytic activities. For example, phosphoglycerate mutase 1, aside from its glycolytic enzymatic activity, could directly interact with α‐smooth muscle actin and modulates cancer cell migration.^45^ In addition to its pyruvate kinase function, the M2 isoform of pyruvate kinase also promotes cyclin D1 expression through binding to β‐catenin.^46^ Moreover, the interaction between lysine‐specific histone demethylase 1 (LSD1) and the transcription factor is necessary for acute myeloid leukaemia survival, instead of the demethylase activity of LSD1.^47^, ^48^ Thus, further studies are necessary to construct an integrated map of the molecular mechanisms of SPR.

**Table 1:** The different effects induced by SPR inhibitors or genetic edition in vitro or in vivo

Biological function	Manipulation	Effect	System	Reference
NO production	N‐acetylserotonin	IL‐1β Induced NO Production	Rat Glomerular Mesangial Cells	36
		IL‐1α + IFN‐γ Induced NO Production	Rat Cardiac Myocytes	35
	Phenprocoumon	IFN‐γ Induced NO Production	Murine Macrophage	37
		IFN‐γ + LPS Induced NO Production		
		IFN‐γ Induced NO Production	Murine Vascular Endothelial Cells	38
		TNF‐α Induced NO Production		
		LPS Induced NO Production		
	Phenprocoumon	Had no Effect on Constitutive Level of NO	Murine Macrophage	37
			Murine Vascular Endothelial Cells	38
	SPR Overexpression	Constitutive Level of NO	Bovine Aortic Endothelial Cells	39
			C57BL6 Mice （In Vivo）	
	SPR Knockdown	Constitutive level of NO	Bovine Aortic Endothelial Cells	
Reactive oxygen species	SPRi3	Impaired the Mitochondrial Function	CD^4+^ T Cells	42
	SPR Knockdown	Impaired the Mitochondrial Function	Dictyostelium Discoideum Ax2	41
	SPR^D257H^	Abolished Sepiapterin Reduction Activity	Lung Epithelial Cells	43
		Had Minimal Effects on Reactive Oxygen Species Production		

## 4 DISTRIBUTION AND REGULATION OF SEPIAPTERIN REDUCTASE

It is reported that sepiapterin reductase has a wide distribution. As mentioned above, SPR has been detected in various species such as *C tepidum*, *Drosophila*, chicken, rat, horse and humans. It has also been found in multiple tissues, including liver, kidney, thymus, brain, spleen, testis and blood.^43^, ^49^, ^50^, ^51^, ^52^, ^53^

**Figure 3:**
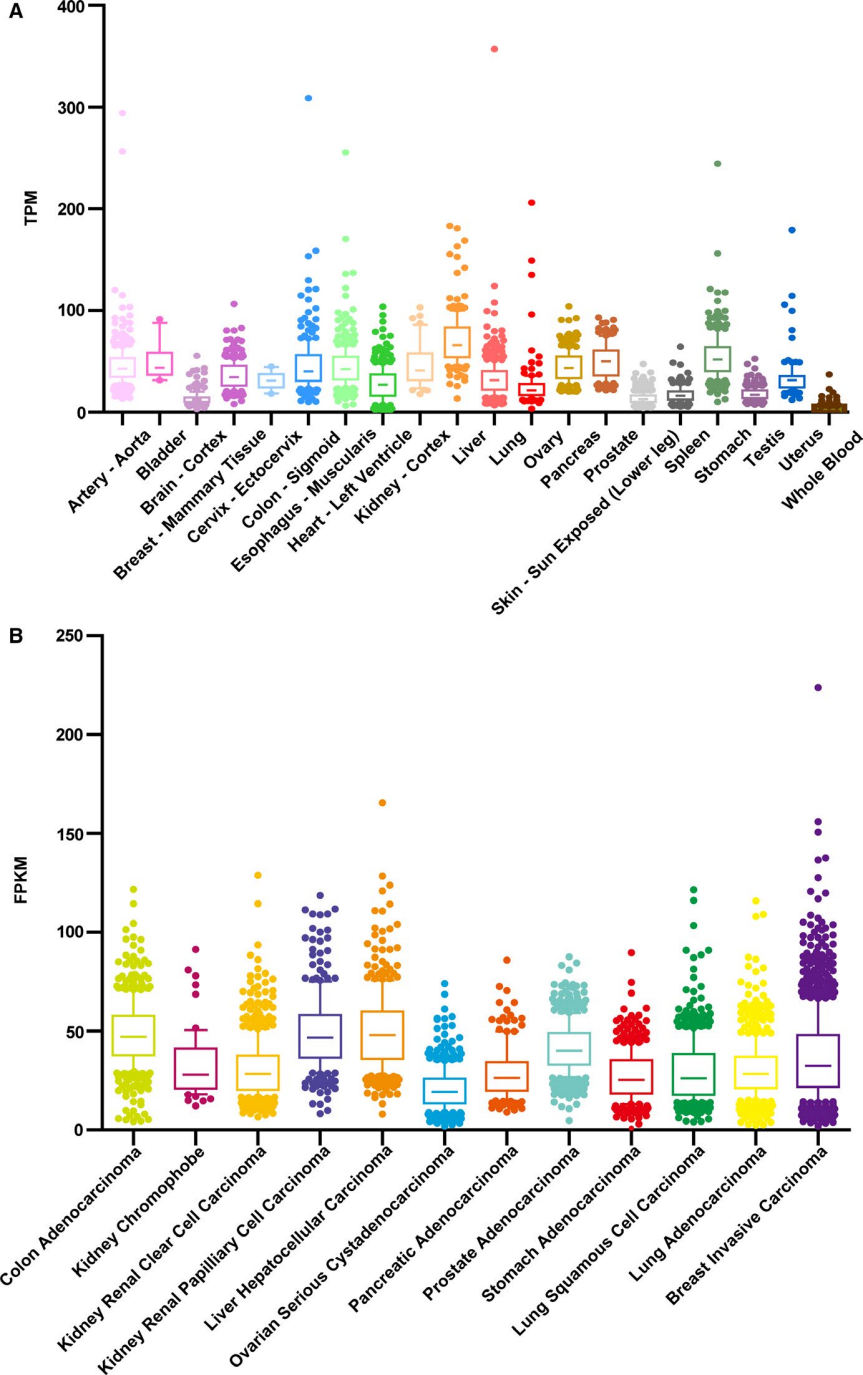
The distribution of sepiapterin reduction in humans. A, The level of the SPR gene in various normal tissues. TPM: Transcripts Per Million. B, The comparison of SPR gene expression in different tumours. FPKM: Fragments per Kilobase Million

Specifically, in rats, SPR activities average 130 and 80 pmol/h/mg in the liver and the erythrocyte fraction of blood, respectively, while no activity is detected in the intestine and muscle.^54^ The enzyme mRNA presents in nearly all the peripheral tissues of goldfish, including intestine and muscle; meanwhile, expression in the brain could be affected by fasting.^55^ Quantitative transcriptomics analysis and microarray‐based immunohistochemistry have been used to analyse the tissue‐specific expression of SPR in a representative set of major human organs and tissues.^56^, ^57^ According to the results, which are presented in a database (gtexportal.org), SPR in normal tissue exhibits relatively high expression levels in the liver, kidney and colon (Figure 3). Furthermore, the distribution of SPR in the human brain has been demonstrated, and the data show that SPR is localized in the pyramidal neurons of the cerebral cortex, in a small number of striatal neurons, and neurons of the hypothalamic and brainstem monoaminergic region and olivary nucleus.^58^ Furthermore, the expression of the SPR gene is also high in liver cancer and colorectal cancer (Figure 3), based on data from The Cancer Genome Atlas dataset (cancergenome.nih.gov). Therefore, SPR might become a clinical biomarker of cancer.

Although the distribution of SPR is wide, no SPR mRNA has been detected in the human NK‐like cell line YT or the murine erythroleukaemic cell line B8/3. Furthermore, the activity and expression of SPR depend on the types of cell lines. For example, in contrast to the liver cell line HepG2, the T‐cell line HuT102 shows lower SPR activity.^49^ However, the enzymatic activity in human T lymphocytes could be continuously stimulated by lectin treatment and achieve a 4‐fold increase.^59^ Moreover, rapid enhancement of SPR activity could be induced in T cells by the synergism of IFN‐γ and IL‐2 rather than by IFN‐γ or by IL‐2 alone.^60^ In the human neuroblastic cell line BE2‐M17, SPR mRNA and protein levels could be down‐regulated by overexpression of wild‐type α‐synuclein.^61^ Lipopolysaccharide treatment has resulted in the up‐regulation of SPR gene expression in the murine neuroblastoma (NB) cell line N1E‐115 ^62^ and the striatum,^63^ while this effect of lipopolysaccharide has not been achieved in the murine locus coeruleus.^64^ Moreover, the serine amino acid residues of rat SPR and hSPR could be phosphorylated by Ca^2+^/calmodulin‐dependent protein kinase II and protein kinase C, but whether phosphorylation has an effect on the activity of SPR or not is controversial.^65^, ^66^ In summary, the study of SPR regulation is rare. To better understand the biological function of SPR and to find new targets for disease therapies, more studies are needed to elucidate the mechanism of SPR regulation.

## 5 SEPIAPTERIN REDUCTASE AND DISEASE

As described above, SPR plays a key role in the de novo biosynthesis of BH_4_. BH_4_ is a key cofactor for a set of metabolic enzymes and is associated with a large number of biological processes, such as monoamine neurotransmitter formation, immune response and pain sensitivity. Therefore, many studies have been conducted to examine the relationship between SPR abnormity and disease.

### 5.1 Sepiapterin reductase and brain dysfunction

Tetrahydrobiopterin is a key cofactor for enzymatic control of the synthesis and secretion of monoamine neurotransmitters, including dopamine and serotonin. Deficiency in the BH_4_ level could result in neurotransmitter responsive disorders that are characterized by motor dysfunction, impaired muscle tone, movement disorders, epileptic seizures and mental retardation. Autosomal dominant mutations in the GTP cyclohydrolase I gene, the rate‐limiting enzyme of BH_4_ synthesis, have been identified as the main cause of these disorders. In 2001, SPD, an autosomal recessive disease, had been discovered first in patients with progressive psychomotor retardation, spasticity and dystonia.^67^, ^68^ However, the diagnosis of SPD was compromised in mild phenotypes because of its variable presenting features and need for a sensitive method of cerebrospinal fluid (CSF) analysis.^69^ Moreover, the pterins profile in CSF is similar to that in patients with defective dihydropteridine reductase. Therefore, only 62 cases of SPD have been reported to date.^69^, ^70^, ^71^, ^72^, ^73^, ^74^, ^75^, ^76^ Commonly, patients exhibit motor and speech delay, axial hypotonia, dystonia, weakness, oculogyric crises and diurnal fluctuation. To avoid misdiagnosis, mutation analysis and activity detection in cultured fibroblasts from patients have been proposed to confirm SPD cases. The manifestations of SPD could be improved by the administration of L‐DOPA or 5‐hydroxytryptophan in combination with carbidopa in the clinic. Interestingly, no case treated with BH_4_, which may, in theory, be of benefit, has shown improvement.^77^

To elucidate the role of SPR in the regulation of BH_4_ homeostasis and neurotransmitter responsive disorders, a mouse strain deficient in the SPR gene has been generated.^78^, ^79^, ^80^, ^81^ SPR‐knockout (SPR^‐/‐^) mice show lower levels of BH_4_, dopamine, serotonin and tyrosine hydroxylase in the brain and liver in contrast to the wild‐type group. In contrast to the data of human patients, the serum phenylalanine level in transgenic mice is significantly increased. One reason for this difference might be that the salvage biosynthetic pathway from 6‐pyruvoyltetrahydropterin to BH_4_ catalysed by 3α‐hydroxysteroid dehydrogenase type 2 (HSDH2) and AR does not function in mice.^32^ Moreover, SPR^‐/‐^ mice exhibit motor dysfunction and developed dwarfism. In addition, most SPR^‐/‐^ mice die within 1‐2 months. The administration of BH_4_ and neurotransmitter precursors could rescue the growth retardation and high phenylalanine levels, but the level of BH_4_ and tyrosine hydroxylase in the brain depends on the method of administration. Furthermore, the tyrosine diet ameliorates the abnormal motor behaviours and enhances mTORC1 activity without affecting dopamine expression in SPR^‐/‐^ mice, suggesting that the mTORC1 signalling pathway in the brain is one of the possible targets in understanding the abnormal motor behaviours related to SPD.^82^, ^83^

Considering that the human SPR gene is located within the region of 2.5 MB mapped to PARK3, which has been identified as an autosomal dominant form of Parkinson's disease (PD), many researchers focus on the relationship between SPR and PD. There is conflicting evidence regarding whether the SPR level is related to PD. Zahra et al^84^ infer that SPR is not the major cause of PD in a Maltese population based on the data from 178 PD cases and 402 control samples. However, the data from Karamohamed et al^85^ and Tobin et al^86^ indicate that SPR mRNA level increases in the brains of PD patients, suggesting a compensatory effect of SPR in PD brain and a role of the SPR in PD pathogenesis. Collectively, several results have suggested that SPR might be associated with various types of brain dysfunction. Nevertheless, further studies are needed to confirm the role and the underlying mechanism of SPR in brain disorder.

### 5.2 Sepiapterin reductase and chronic pain

The data of gene expression profiling in the dorsal root ganglion in three different models of neuropathic pain showed that three of the enzymes, including GTPCH, SPR and quinoid dihydropteridine reductase, which are critical to the control of intracellular levels of BH_4_, were highly regulated within injured sensory neurons.^87^ Although GTPCH, an obligate rate‐limiting enzyme in BH_4_ synthesis, plays an important role in chronic pain,^88^, ^89^, ^90^ the therapeutic index for GTPCH inhibitors might be relatively limited due to the side effects induced by the dramatic reduction of BH_4_.^91^, ^92^ In contrast to GTPCH, SPR could be bypassed in the biosynthesis of BH_4_. Furthermore, N‐acetylserotonin (NAS), an inhibitor of sepiapterin reductase, could significantly reduce chronic pain in the spared nerve injury model and the paw inflammation model through inhibiting BH_4_ production.^87^ Therefore, SPR has become a more attractive drug target for chronic pain, and many studies have focused on the development of SPR inhibitors.^91^, ^93^, ^94^ In particular, Latremoliere et al^91^ developed a new SPR inhibitor—SPRi3—and proved that SPR inhibition was a viable approach for normalizing neuropathic and inflammatory pain hypersensitivity without unacceptable side effects. In summary, the enzymatic activity of SPR could regulate chronic pain and is a valuable target for the development of analgesics.

### 5.3 Sepiapterin reductase and cardiovascular disease

Nitric oxide, synthesized by three nitric oxide synthases, is an important regulator of vascular tone. All NOS isoforms require BH_4_ as cofactor for catalytic activity, reiterating that SPR might be implicated in cardiovascular disease. Many studies have demonstrated that cytokine‐induced NO production in murine macrophages and endothelial cells could be abolished by SPR inhibitors.^35^, ^36^, ^37^, ^38^ Gao and coworkers^39^ proved that the overexpression or depletion of SPR could affect NO production in endothelial cells and NO‐dependent vasorelaxation in vivo. Furthermore, the endothelium‐specific SPR deficiency in deoxycorticosterone acetate‐salt hypertensive mice suggests the importance of SPR in maintaining normal blood pressure.^95^ Nevertheless, aortic endothelial SPR expression is unaffected in angiotensin II‐induced hypertensive mice ^95^ and mice with pulmonary hypertension induced by bleomycin.^96^ Moreover, the therapeutic effects of sepiapterin, a substrate of SPR, in hypertension depend on the level of SPR in the arteries. Therefore, strategies specifically targeting SPR might be necessary for restoring NOS function in different types of hypertension. Recently, the cardiovascular function of SPR gene‐disrupted mice has been analysed.^97^ After weaning, SPR^‐/‐^ but not SPR^+/−^ adult mice suffered from hypertension with fluctuation and bradycardia due to the decrease in endothelium‐dependent relaxation. At the same time, the imbalance of the sympathetic and parasympathetic nervous systems found in the SPR^‐/‐^ mice might contribute to cardiovascular instability. Besides, the SPR inhibitor NAS could completely inhibit the sepiapterin‐stimulated tube formation of bovine aortic endothelial cells in vitro.^98^ Although the key role of SPR in cardiovascular disease has been proposed, the underlying mechanism remains unclear. Further studies are needed to answer the remaining questions.

### 5.4 Sepiapterin reductase and cancer

In recent years, the role of BH_4_ in the development of cancer has attracted increasing interest. As the terminal enzyme in the BH_4_ biosynthetic pathway, SPR has also been studied in tumour progression. Tanaka et al^99^ find that N‐acetylserotonin, an inhibitor of SPR, could inhibit DNA synthesis and initiate the differentiation of erythroleukaemia (MEL) cells, which is reversed by restoration of cellular BH_4_ level with sepiapterin. However, results contradicting this suggestion have also been reported. It has been demonstrated that none of the SPR inhibitors, including NAS, dicumarol, ethacrynic acid and N‐chloroacetyldopamine, could induce the differentiation of MEL cells. Additionally, inhibition of the MEL cell growth rate induced by these inhibitors could not be prevented by the addition of BH_4_ or BH_4_‐related pterins.^100^ In MOLT‐4 T‐lymphoblastic leukaemia cells and MCF‐7 breast cancer cells, the depletion of BH_4_ using an SPR inhibitor does not affect cell proliferation.^101^

Remarkably, Kaplan‐Meier analysis of 88 human NB tumours indicates that high SPR gene expression is significantly correlated with poor survival prognosis. In vitro, SPR‐knockdown results in a significant decrease in the proliferation of NB cells, which presumably is relevant to the interaction between SPR and ornithine decarboxylase, which is a regulator of cell division, proliferation and apoptosis.^102^ Sulfasalazine (SSZ), which has been identified as an SPR inhibitor, could inhibit the growth of NB cells and produce synergistic antiproliferative effects in combination with alpha‐difluoromethylornithine.^103^ Furthermore, oral/intraperitoneal SSZ co‐administration resulted in measurable inhibition of NB tumour growth in vivo.^104^ Recently, it is reported that SPR is required for the proliferation of mature T cells in vitro and in vivo and that the inhibition of cancer induced by SPR inhibitors links to the immunosuppressive tumour environment.^42^ All of these studies provide potential targets for cancer therapy, although further research illuminating the role of SPR in cancer progression and the mechanism underlying the regulation is needed.

## 6 DRUGS TARGETING SEPIAPTERIN REDUCTASE

Given the pivotal role of SPR in various diseases, many compounds targeting SPR have been found. Valproic acid, a first‐line drug in the treatment of bipolar disorder, is proven to have a strong up‐regulatory effect on SPR at the gene and protein levels because of its histone deacetylase (HDAC) inhibitory activity.^105^ Two other HDAC inhibitors, trichostatin A and sodium butyrate, could also up‐regulate SPR expression. In addition, the DNA methylation inhibitor 5'‐aza‐deoxycytidine induces a significant increase in SPR levels, with an over 8‐fold increase at 1 μmol/L.^105^

**Figure 4:**
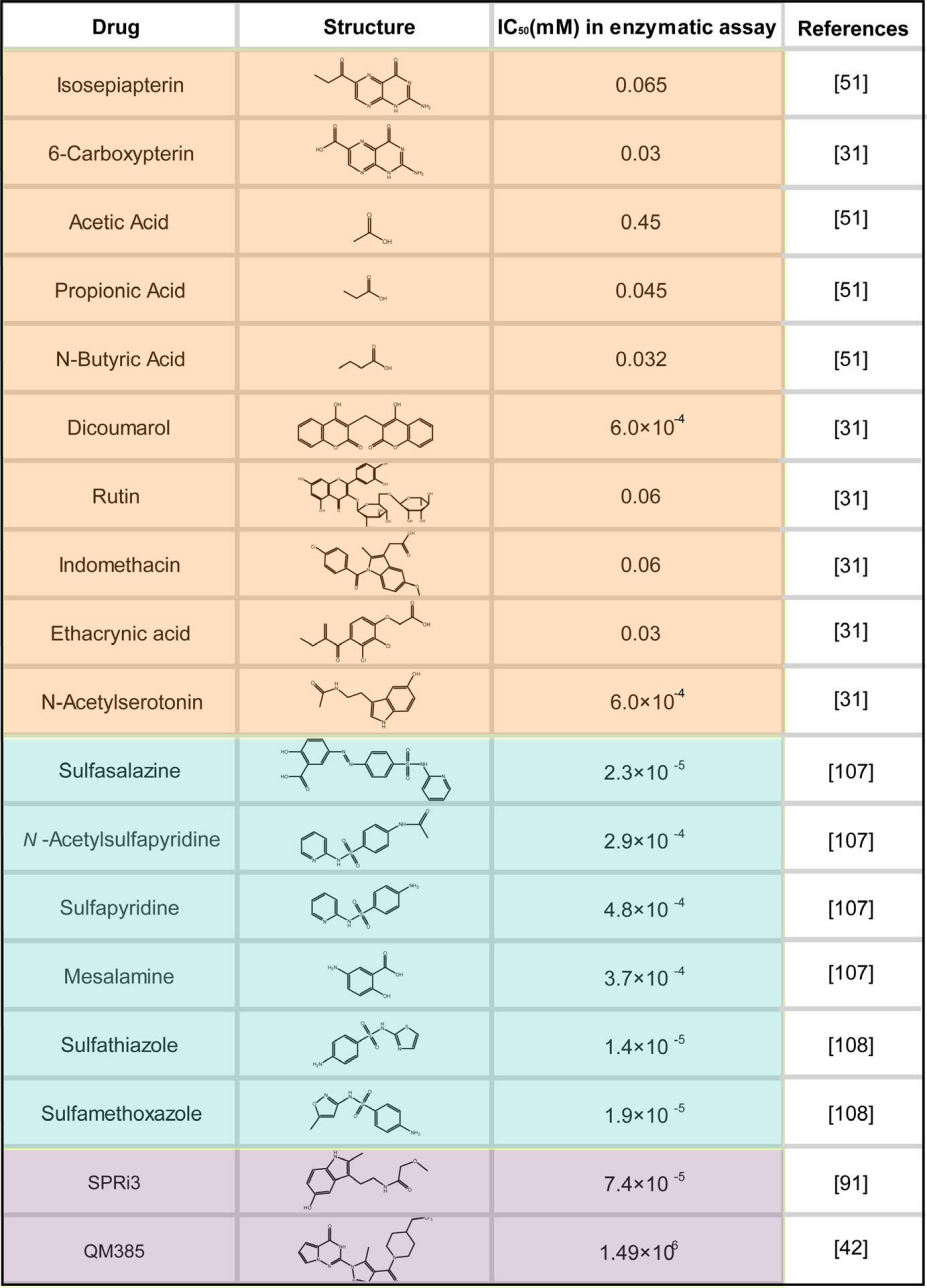
The chemical structure of SPR inhibitors and their IC_50_ values in an enzymatic assay

Various effective inhibitors of SPR have been identified as exhibited in Figure 4. The process of inhibitor development is divided into three stages. First, it is found that some sulphhydryl reagents, such as N‐ethylmaleimide, and unconjugated pteridines, including isosepiapterin and aliphatic monocarboxylic acids, can inhibit the activity of SPR.^51^ Based on the fact that SPR belongs to a series of enzymes that have been classified as aldo‐keto reductases, many inhibitors against general aldo‐keto reductases, for example dicoumarol, rutin, indomethacin and ethacrynic acid, have been studied and proven to inhibit SPR enzyme activity towards either a carbonyl compound of a non‐pteridine derivative or sepiapterin as substrate.^31^, ^101^, ^106^ The second stage begins with the introduction of a methodology combining yeast three‐hybrid screening with affinity chromatography. Applying this approach, Chidley et al^107^ and Haruki et al^108^ found that sulphonamides, such as sulphathiazole, sulphamethoxazole, SSZ and its metabolites, sulphapyridine and mesalamine, could inhibit the SPR enzyme activity with high potency. Moreover, the binding mode of sulphonamides to SPR has been elucidated by crystallographic studies. The results show that SPR, NADP^+^ and sulphonamides form a ternary complex, in which the sulpha drugs form specific hydrogen bonds with active site residues Ser157, Tyr170 and Asp257 of SPR.^108^, ^109^ Finally, due to the limited bioavailability, low potency and complex metabolism of sulphonamides, more potent SPR inhibitors, SPRi3^91^ and QM385,^42^ have been developed. Compared to sulphonamides, the third SPR inhibitors show a higher affinity to human SPR and higher potency in reducing BH_4_ synthesis in cells. Recently, using NMR and X‐ray supported fragment screening, Alen et al^93^ obtained a new compound with good physicochemical and in vitro ADME properties that could function as a candidate for follow‐up hit‐to‐lead optimization. All of these drugs provide possible therapies for various diseases; however, considering the toxicity and bioavailability of these inhibitors, new inhibitors should be developed. Otherwise, the different effects induced by SPR depletion and enzyme inhibition suggest that the non‐enzymatic function of SPR provides a potential direction for the development of SPR inhibitors.

## 7 FUTURE DIRECTIONS

The important biological function of SPR has been demonstrated by using molecular biological techniques, ranging from protein X‐ray structure determinations to mutagenesis studies. Furthermore, a set of drugs targeting SPR have been identified, which makes the studies of the SPR enzymatic activity easier. In addition, the discovery of patients with SPD has provided new insight into the role of SPR in disease and provided potential therapeutic strategies and biomarkers for brain dysfunction, chronic pain, cardiovascular disease and cancer. However, for obtaining an integrated map of the molecular biology of SPR, many questions remain to be solved, such as the following. (a) What causes the different effects induced by SPR inhibitors or SPR knockdown? Does the non‐enzymatic function of SPR work or not? What is the molecular mechanism modulating the function independent of the enzymatic activity? (b) What is the specific regulation pathway of SPR expression and the degradation pathway of the protein? Could drugs be developed based on these pathways? (c) Why do patients with SPD not show improvement after BH_4_ treatment? Is this lack of improvement related to the non‐enzymatic activity or not? (d) How does SPR regulate the progression of NB? Does SPR affect other types of tumours, such as lung cancer and gastric carcinoma? (e) Could compounds targeting the non‐enzymatic activity of SPR treat diseases and produce reduced side effects compared to the inhibitors of enzymatic function or not?

## CONFLICT OF INTEREST

The authors confirm that there are no conflicts of interest.

## AUTHOR CONTRIBUTION

**Yao Wu:** Conceptualization (equal); Data curation (lead); Investigation (lead); Resources (equal); Writing‐original draft (lead). **Peng Chen:** Investigation (supporting); Resources (equal); Writing‐original draft (supporting); Writing‐review & editing (equal). **Li Sun:** Funding acquisition (supporting); Writing‐review & editing (equal). **Shengtao Yuan:** Funding acquisition (supporting); Writing‐review & editing (equal). **Zujue Cheng:** Funding acquisition (supporting); Writing‐review & editing (equal). **Ligong Lu:** Funding acquisition (supporting); Writing‐review & editing (equal). **Hongzhi Du:** Conceptualization (equal); Funding acquisition (supporting); Supervision (equal); Writing‐review & editing (lead). **Meixiao Zhan:** Funding acquisition (lead); Supervision (equal); Writing‐review & editing (equal).

## Data Availability

Data sharing is not applicable to this article as no new data were created or analysed in this study.
